# Selenium‐Enriched *Cordyceps militaris* Polysaccharides Alleviate Insulin Resistance in HepG2 Cells by Regulating the PI3K/AKT/GLUT4 Signaling Pathway

**DOI:** 10.1002/fsn3.70246

**Published:** 2025-05-10

**Authors:** Jiali Chen, Xiumin Zhang, Yanting Chang, Na Zhang, Xiqing Yue, Mohan Li, Wentao Jia

**Affiliations:** ^1^ College of Food Science Shenyang Agricultural University Shenyang China; ^2^ College of Food Engineering Harbin University of Commerce Harbin China; ^3^ Beijing Academy of Food Sciences Beijing China; ^4^ Jiufukang Industrial Co., Ltd. Shengzhou China

**Keywords:** anti‐insulin resistance, antioxidant activity, *Cordyceps militaris*, PI3K/AKT/GLUT4 signaling pathway, polysaccharides, selenium

## Abstract

Metal ion‐rich *Cordyceps militaris* polysaccharides (CMP) are obtained by biotransformation and chemical synthesis. The physicochemical properties and bioactivity of selenium‐enriched CMP (Se‐CMP) obtained using biotransformation remain unclear. Here, selenium nanoparticles were used to produce Se‐CMP. Total sugars, molecular weight, monosaccharide composition, and galacturonic acid and Se contents of Se‐CMP and CMP were determined. The Se content of Se‐CMP was 5.11 ± 0.81 μg/g. The Se‐CMP is composed of glucosamine hydrochloride, xylose, arabinose, galactose, mannose, and glucose in a molar ratio of 0.001: 0.036: 0.022: 0.133: 0.150: 0.659. Se‐CMP was structurally characterized using Fourier transform infrared spectroscopy and circular dichroism spectroscopy. Se‐CMP inhibited α‐glucosidase activity (half‐maximal inhibitory concentration 0.474 mg/mL). Se‐CMP also increased glucose uptake and glycogen synthesis in insulin‐resistant HepG2 cells by activating the PI3K/AKT/GLUT4 signaling pathway. It also effectively reduced the level of reactive oxygen species in insulin‐resistant HepG2 cells while increasing the activity of antioxidant enzymes, including catalase, glutathione peroxidase, and superoxide dismutase, reducing oxidative damage. Se‐CMP has significantly alleviated insulin‐resistant properties and may be a natural anti‐IR source for managing Type II diabetes mellitus.

## Graphical abstract

The structure of selenium‐enriched *Cordyceps militaris* polysaccharides has been characterized. Demonstrating the effects of selenium‐enriched *Cordyceps militaris* polysaccharides in alleviating insulin resistance and reducing oxidative stress. Investigating the mechanisms by which selenium‐enriched *Cordyceps militaris* polysaccharides alleviate insulin resistance.

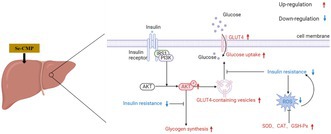

## 1 Introduction

Diabetes mellitus (DM) is a globally prevalent chronic disease. DM is primarily attributed to insulin resistance (IR) or insufficient insulin (Zhang et al. 2019a). Current data from the International Diabetes Federation indicate that the global population with diabetes will reach 537 million by 2023, with projections suggesting an increase of approximately 1.31 billion by 2050. Over 90% of patients are diagnosed with Type II diabetes mellitus (T2DM) (Qian et al. 2020). IR is a significant factor in the onset of T2DM. IR commonly occurs in individuals with metabolic disorders (Cook et al. 2015). The therapeutic landscape for T2DM primarily includes biguanide, thiazolidinedione, and sulfonylurea drugs. Challenges associated with these pharmacological agents include complex dosing schedules, side effects, high costs, and potential toxicity. Accordingly, the research focus has shifted toward identifying natural edible products with low toxicity and high efficacy as potential alternatives for managing T2DM (Zhang et al. 2019b). Numerous studies have demonstrated that many medicinal food homology materials have preventive or therapeutic hypoglycemic efficacy and are non‐toxic, with few or no adverse effects (Xia and Xiao 2021). Examples include *Cordyceps militaris* (Zhang et al. 2006), *Polygonatum sibiricum* (Luo et al. 2020), and *Astragalus* (Zhu et al. 2016). Furthermore, the incorporation of trace elements, especially selenium (Se) (Duan et al. 2022; Xiao et al. 2019), into food products through biotransformation or synthesis methods further alleviates IR and enhances these hypoglycemic effects. This synergistic approach highlights the potential of combining traditional medicinal foods with trace elements to develop effective dietary interventions for the management of blood glucose. Se is an indispensable trace element for animals and humans. It plays a crucial role in the body's defense against a range of health conditions, including aging, diabetes, oxidative stress (OS), and enhanced immune function (Hou et al. 2016). As an insulin mimetic, Se has antidiabetic effects (Ogawa‐Wong et al. 2016). However, Se cannot be synthesized endogenously and must be obtained from dietary sources. Notably, billions of people worldwide reside in regions with Se deficiency, particularly in China, New Zealand, and Europe (Chen et al. 2021). Given this deficiency, supplementation with exogenous Se is critical to meet bodily requirements. Se supplements can be broadly categorized into inorganic and organic forms. Owing to its high absorption rate, low toxicity, and suitability for prolonged use, organic Se offers significant advantages over inorganic Se. Among the organic Se compounds, Se polysaccharides are distinguished by their enhanced stability and biological activity (Zhou et al. 2018). These distinctions emphasize the importance of selecting the appropriate Se supplementation to optimize health benefits.

*C. militaris* , also recognized as North *C. sinensis* , is an edible and medicinal fungus that was approved by the Ministry of Health of the People's Republic of China as a new food resource in 2009. Currently, *C. militaris* is cultivated artificially and has a significant market share in China for both edible and medicinal mushrooms (Zhao et al. 2024). It contains a spectrum of bioactive components, including cordycepin, polysaccharides, proteins, and cordycepic acid, which exhibit anticancer (Tuli et al. 2013), immunomodulatory (Fan et al. 2021), gut microbiota‐regulating (Jiang et al. 2024), anti‐inflammatory (Guo et al. 2024), and hypoglycaemic properties (Ni et al. 2024). Polysaccharides are particularly significant bioactive components of *C. militaris* and have diverse pharmacological properties (Miao et al. 2022). Recent studies have extensively explored the chemical selenylation of these polysaccharides to enhance their biological effectiveness (Liu et al. 2017). Despite substantial progress in chemical modification techniques, the application of biotransformation processes to produce Se‐enriched CMP (Se‐CMP) and their physicochemical properties and ability to alleviate IR remain underexplored.

This study was undertaken to determine the physicochemical and structural characteristics of CMP and Se‐CMP and to establish IR Human hepatoma cells (HepG2) to explore the potential mechanisms of anti‐IR of Se‐CMP. The ultimate goal is to aid individuals with IR by devising a natural dietary alternative with minimal side effects, thereby assisting in lowering blood glucose levels.

## 2 Materials and Methods

### 2.1 Materials and Reagents

The enrichment process for obtaining Se‐CMP from Se nanoparticles has been previously described (Hu et al. 2019). HepG2 cells were obtained from the Cell Bank of the Chinese Academy of Sciences (Shanghai, China). Dulbecco's modified Eagle's medium high glucose medium was purchased from HyClone (Logan, Utah, USA). Fetal bovine serum (FBS), 0.25% tryptase‐EDTA, and pre‐stained protein molecular weight standard were purchased from Thermo Fisher Scientific (China) Ltd. (Shanghai, China). Dimethyl sulfoxide (cell culture grade) and recombinant human insulin were purchased from Beijing Priority Genetic Technology Co. Ltd. (Beijing, China). The sodium dodecyl sulfate‐polyacrylamide gel electrophoresis (SDS‐PAGE) gel preparation kit (Cat. No. WLA013), ECL luminescent liquid (Cat. No. WLA003), and 3‐(4, 5‐dimethylthiazolyl‐2)‐2, 5‐diphenyltetrazolium bromide (MTT, Cat. No. WLA021) kit were purchased from Wanlianbio (Shenyang, China). PBS buffer (10×, pH 7.2–7.4) was purchased from Collins (Shanghai, China). Monosaccharide standards, α‐Glucosidase, and metformin (Met) were purchased from Sigma‐Aldrich (St. Louis, MO, USA). Skimmed Milk Powder was purchased from Yili Co. (Inner Mongolia, China). PVDF membranes were purchased from Millipore (MA, USA) All other chemicals and reagents used were of analytical grade.

### 2.2 Extraction and Isolation of Polysaccharides

The extraction of CMP and Se‐CMP was performed as previously described (Hu et al. 2019), with slight modification. Briefly, the samples were extracted with distilled water at a ratio of 1:10 (g/v), sonicated (26 min), macerated (80°C, 120 min), and centrifuged (50°C, 15 min, 4000 rpm). The extracts were centrifuged, concentrated, precipitated with anhydrous ethanol, and lyophilized. Proteins were deproteinized by the Sevage method (Malinowska et al. 2018), and organic solvents were removed by spin evaporation at 60°C. CMP and Se‐CMP were obtained by dialysis in a dialysis bag with a retention capacity of 3.5 kDa at 4°C for 72 h.

### 2.3 Characterization of CMP and Se‐CMP

#### 2.3.1 Determination of Purity and Contents of Total Sugars, Se, and Galacturonic Acid

The total sugars content of CMP and Se‐CMP was determined using the phenol‐sulfuric acid method, with glucose serving as a standard. The Se content was determined as previously described (Luo et al. 2021) with slight modifications using microwave digestion pretreatment, followed by inductively coupled plasma mass spectrometry (ICP‐MS). To ascertain the content of galacturonic acid, 1 mL of the sample (50 μg/mL) was collected, and the optical density at 523 nm was determined by adding sodium tetraborate‐sulfuric acid solution and carbazole solution (Zayed et al. 2020). The purity of the polysaccharides was determined by ultraviolet (UV) spectroscopy in the wave number range of 200–400 nm.

#### 2.3.2 Molecular Weight Determination

High‐performance gel permeation chromatography using a model 1515 instrument (Waters, USA) was used to measure the molecular weights of CMP and Se‐CMP (Hongxia 2017). The mobile phase was a 0.05 M NaCl solution. Dextrans with different molecular weights (1, 5, 12, 25, 50, 80, and 670 kDa) were used as standards to construct the molecular weight calibration curve.

#### 2.3.3 Analysis of Monosaccharide Composition

High‐performance anion exchange chromatography using an ICS5000 instrument (Thermo Fisher Scientific, USA) was used to determine the monosaccharide compositions of CMP and Se‐CMP (Zhang et al. 2018b).

#### 2.3.4 Fourier Transform Infrared Spectroscopy (FT‐IR)

CMP and Se‐CMP (2 mg each) were mixed with 200 mg of KBr powder and pressed into tablets. The infrared spectra were collected at wavelengths ranging from 4000 to 400 cm^−1^.

#### 2.3.5 Circular Dichroism (CD)

CMP and Se‐CMP were each configured into a 1 mg/mL solution, and the sample solution was measured using a CD spectrometer at 100 nm/min, and data were collected in the range of 195–260 nm.

### 2.4 In Vitro Assays of Se‐CMP to Alleviate IR

#### 2.4.1 α‐Glucosidase Inhibitory Activity Analysis

The α‐glucosidase inhibitory activity of Se‐CMP was determined as previously described (Ou et al. 2023).

#### 2.4.2 Cell Culture and Treatment

HepG2 cells were cultured with Dulbecco's modified Eagle's medium containing 10% FBS at 37°C in an atmosphere of 5% CO_2_. Cells were treated with insulin at a concentration of 10^−6^ mol/L for 24 h to establish an IR cell model. The normal control group consisted of HepG2 cells that were not exposed to insulin. In the positive control group, IR cells were treated with 10 mM metformin for IR cells. In the experimental group, IR cells were treated with Se‐CMP (1000, 800, 600, 400, 200, or 100 μg/mL).

#### 2.4.3 Cell Viability and Cell Morphology Analyses

IR HepG2 cells were incubated with Se‐CMP (1000, 800, 600, 400, 200, or 100 μg/mL) and Met (10 mM) for 48 h. Cell viability was determined using the MTT method as previously described (Wang et al. 2020). The cell morphology was observed by inverted fluorescence microscopy at 200× magnification.

#### 2.4.4 Glucose (Glu) Consumption

IR HepG2 cells were incubated with Se‐CMP (1000, 800, 600, 400, 200, or 100 μg/mL) and Met (10 mM) for 48 h. Cells were collected (300 *g*) and centrifuged (10 min) to remove the precipitate. The content of Glu was determined using a glucose assay kit (Cat. No. WLA134, Wanleibio, Shenyang, China) according to the manufacturer's instructions.

#### 2.4.5 Intracellular Glycogen Content

IR HepG2 cells were incubated with Se‐CMP (1000 μg/mL) and Met (10 mM) for 48 h. Cells were collected (5 × 10^6^–1 × 10^7^) in a centrifuge tube. Following centrifugation, the supernatant was discarded. The cells were resuspended in 0.75 mL of extraction solution in a 10 mL test tube, broken by ultrasonication, placed in a boiling water bath, and boiled for 20 min. During the boiling, the tube was shaken once every 5 min to ensure sufficient mixing. The samples were cooled and then diluted to 5 mL with distilled water, mixed well, and centrifuged (25°C, 10 min, 8000 × *g*). The supernatant was extracted, and the liver glycogen content was determined using the BCA protein concentration assay kit (Cat. No. WLA004, wanleibio, Shenyang, China) and glycogen content assay kit (Cat. No. bc0340, Solarbio, China) according to the manufacturer's instructions.

#### 2.4.6 Reactive Oxygen Species (ROS)

The intracellular level of ROS was determined as previously described (Lv et al. 2025). Briefly, IR HepG2 cells were incubated with Se‐CMP (1000 μg/mL) and Met (10 mM) for 48 h. One milliliter of a 1:000 dilution in the medium of DCFH‐DA was added and mixed, and the cells were incubated at 37°C for 20 min. The tubes were inverted every 5 min. The cells were washed three times using PBS. Following the final wash, the cells were resuspended in PBS, and a flow cytometry assay was performed.

#### 2.4.7 Measurements of Superoxide Dismutase (SOD), Catalase (CAT), and Glutathione Peroxidase (GSH‐Px) Activities

IR HepG2 cells were incubated with Se‐CMP (1000 μg/mL) and Met (10 mM) for 48 h. The cells were resuspended in PBS, lysed by sonication in an ice bath, and centrifuged at 1500 × *g* for 10 min. Each supernatant was collected for the assay. Protein was determined, and the intracellular activities of CAT, GSH‐Px, and SOD were determined using BCA protein concentration assay kits (Cat. No. WLA004, Wanleibio, Shenyang, China), SOD assay kits (Cat. No. WLA110, Wanleibio, Shenyang, China), CAT assay kits (Cat. No. A007, Nanjing Built, Nanjing, China), and GSH‐Px assay kits (Cat. No. WLA107, Wanleibio, Shenyang, China) according to the manufacturer's instructions.

#### 2.4.8 Western Blot

IR HepG2 cells were incubated with Se‐CMP (1000 μg/mL) and Met (2 mM) for 48 h. Western blotting was performed as previously described with slight modification (Qian et al. 2020). Proteins were separated by 10% SDS‐PAGE. Target proteins were detected using primary antibodies against phosphorylated phosphatidylinositol 3‐kinase (p‐PI3K) (Cat. No. AF3241, Affinity, USA), protein kinase B (AKT, Cat. No. WL0003b), phosphatidylinositol 3‐kinase (PI3K, Cat. No. WL02240), phosphorylated protein kinase B (p‐AKT, Cat. No. WLP001a), glucose transporter 4 (GLUT4, Cat. No. WL02425), and β‐actin (Cat. No. WL01372) (all from Wanlianbio, Shenyang, China).

#### 2.4.9 Immunofluorescence Staining for Glucose Transporter Type 4 (GLUT4)

IR HepG2 cells were incubated with Se‐CMP (1000 μg/mL) and Met (2 mM) for 48 h. Cells were fixed in 4% paraformaldehyde (15 min). Triton X‐100 (0.1%) was added dropwise to completely cover the cells, followed by incubation (25°C, 30 min). Bovine serum albumin (1%) was then added dropwise, followed by incubation (25°C, 15 min). During 16 h at 4°C of primary antibody incubation, the cells were dropwise treated with fluorescent secondary antibody in the dark (1 h). Cell nuclei were stained with DAPI, and the staining effect was observed under a fluorescence microscope.

### 2.5 Statistical Analysis

At least three independent runs were performed for each cell culture experiment. Every value is expressed as $\bar{x}$ ± s. Analysis of variance was used to assess the experimental data, with *p* < 0.05 and considered statistically significant. SPSS Statistics for Windows, version 27.0 (IBM, USA), was used for statistical analysis.

## 3 Results

### 3.1 Total Sugars, Se, Galacturonic Acid Contents, and Purity of CMP and Se‐CMP

**FIGURE 1:**
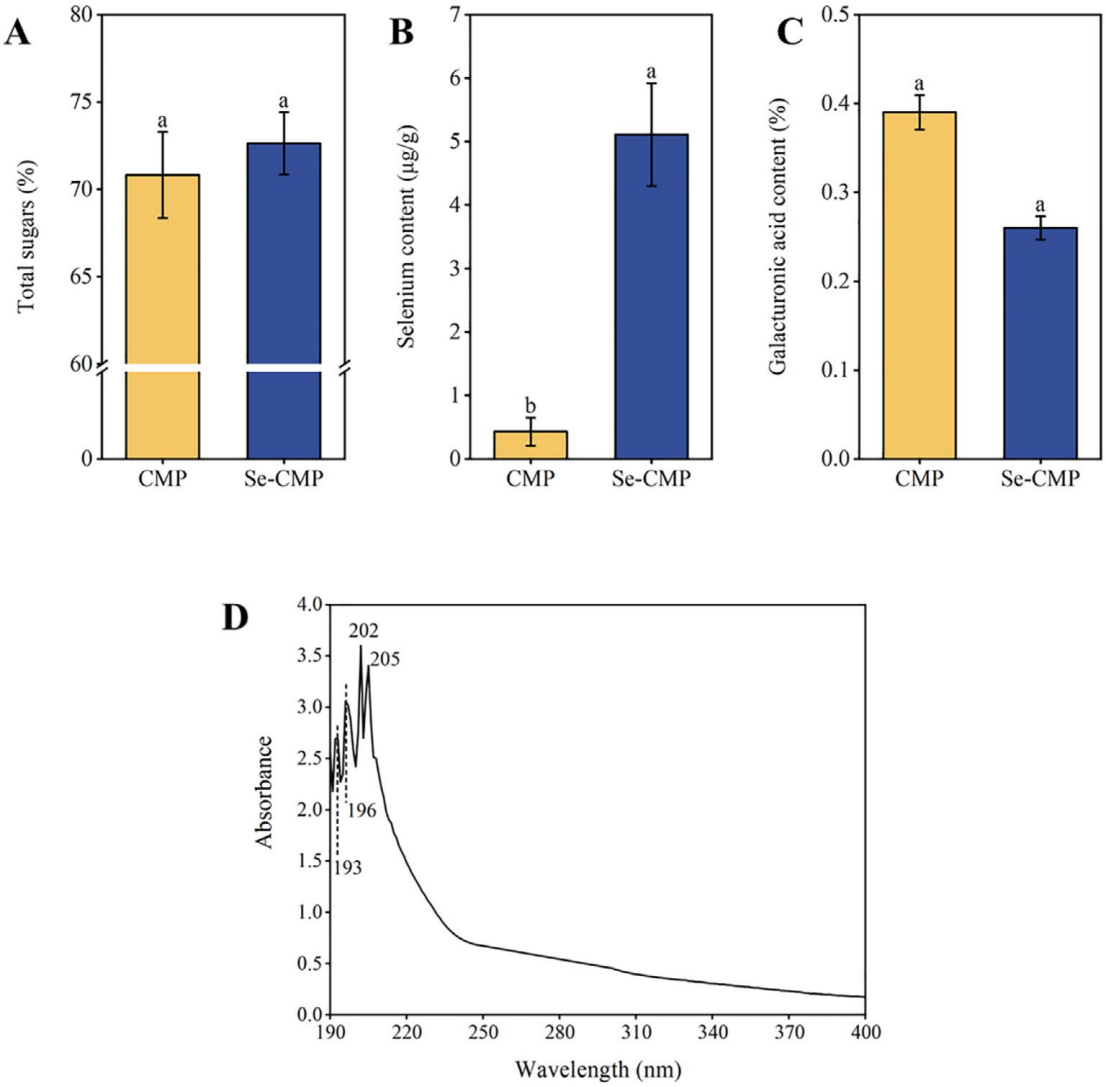
Contents of total sugars (A), Se (B), and galacturonic acid (C) of CMP and Se‐CMP, and UV spectrum of Se‐CMP (D). CMP: *Cordyceps militaris* polysaccharides; Se, selenium; Se‐CMP, selenium‐enriched *Cordyceps militaris* polysaccharides. Data marked with different letters are significantly different at *p* < 0.05.

The content of total sugars was 70.82% ± 2.46% for CMP and 72.63% ± 1.77% for Se‐CMP (Figure 1A). ICP‐MS analysis revealed that the Se content of Se‐CMP was significantly higher than the content in CMP (5.11 ± 0.81 vs. 0.43 ± 0.22 μg/g; *p* < 0.05), confirming the successful enrichment of Se in CMP (Figure 1B). Similarly, CMP and eCMP produced through biotransformation exhibited a similar pattern 0.10 ± 0.01 versus 5.14 ± 0.06 μg/g Se, respectively (Yu et al. 2021). These findings demonstrate that Se can be effectively incorporated into polysaccharides through biotransformation.

The galacturonic acid content in CMP and Se‐CMP was 0.39% ± 0.46% and 0.26% ± 0.54%, respectively. These values suggest that both CMP and Se‐CMP are neutral polysaccharides owing to their low galacturonic acid concentrations (Figure 1C). Additionally, the UV spectra of Se‐CMP showed no absorption peaks (AP) at 260 and 280 nm (Figure 1D), demonstrating the absence of nucleic acids and proteins in the Se‐enriched polysaccharides. The lack of nucleic acid and protein contamination further confirmed the purity of the Se‐CMP preparation.

### 3.2 Molecular Weight and Monosaccharide Composition of CMP and Se‐CMP

**FIGURE 2:**
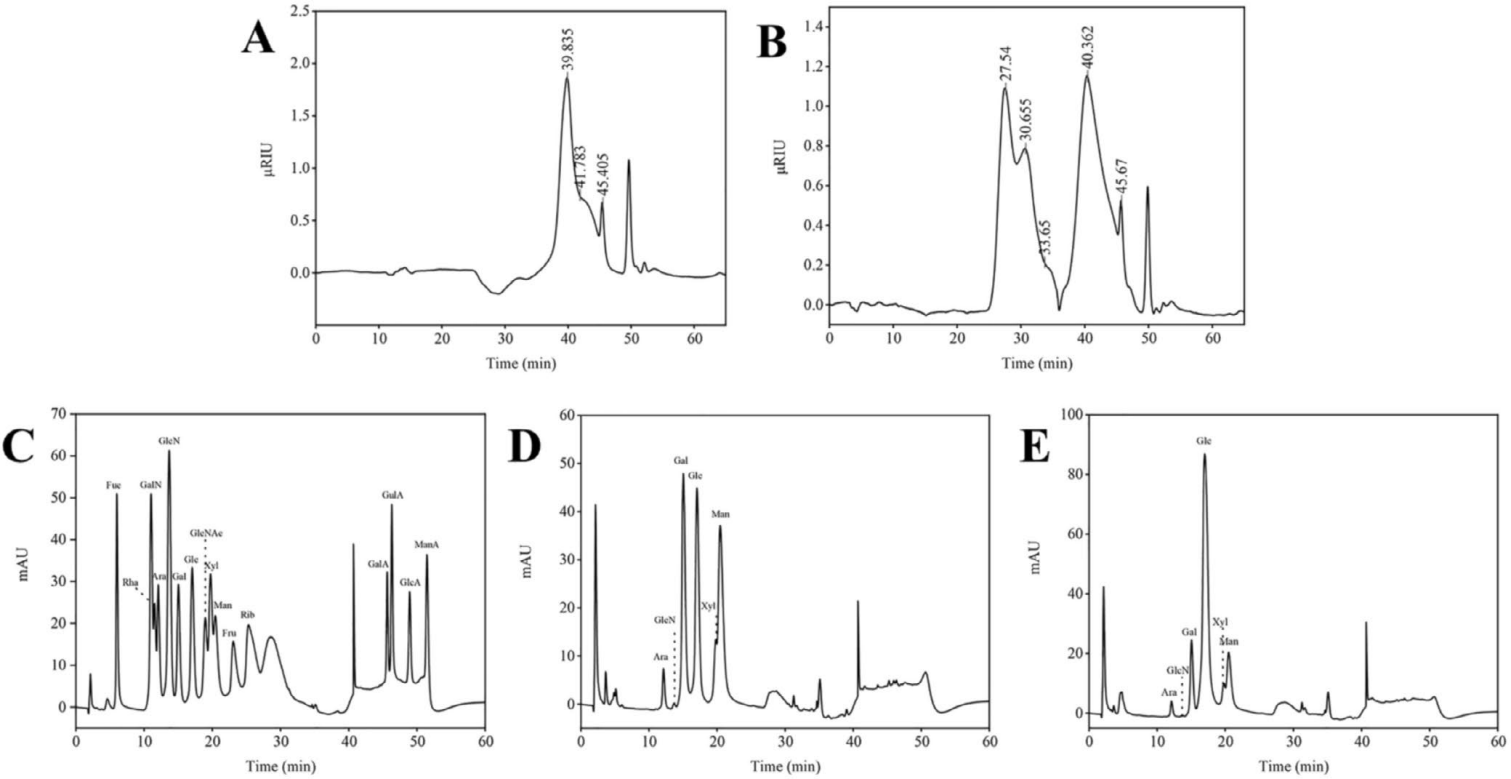
High‐performance gel permeation chromatograms of CMP (A) and Se‐CMP (B), high‐performance anion exchange chromatograms of standard monosaccharides (C), and the monosaccharide composition of CMP (D) and Se‐CMP (E). CMP, *Cordyceps militaris* polysaccharides; Se‐CMP, selenium‐enriched *Cordyceps militaris* polysaccharides.

The molecular weight distribution results, depicted in Figure 2A,B, revealed that three molecular weight fractions for CMP: 26,881 Da (67.12% of the total), 12,749 Da (24.85%), and 3185 Da (8.03%). In contrast, Se‐CMP exhibited a broader range, with five molecular weight distributions: 2,979,189 Da (23.66%), 903,802 Da (21.24%), 287,081 Da (3.16%), 21,968 Da (46.41%), and 2878 Da (5.53%). The number of fractions and molecular weights of the polysaccharides increased with Se enrichment (regression equation, lgMw = −0.1663x + 11.054; *R* ^2^ = 0.9950; Tables S1 and S2), showed that the number of fractions and molecular weights of the polysaccharides increase with selenium enrichment.

Further analysis of the monosaccharide composition, presented in Figure 2C–E, showed that both CMP and Se‐CMP comprised six monosaccharides: glucosamine hydrochloride, xylose, arabinose, galactose, mannose, and glucose. The respective molar ratios for CMP were 0.001: 0.059: 0.041: 0.313: 0.360: 0.226. The respective molar ratios for Se‐CMP were 0.001: 0.036: 0.022: 0.133: 0.150: 0.659 (Table S3). The findings indicate that although the types of monosaccharides present in CMP and Se‐CMP were consistent, their ratios varied significantly. In both Se‐CMP and CMP, galactose, mannose, and glucose are the major monosaccharides, which is in agreement with the summary results of Jixian Zhang et al. on the monosaccharide composition of CMP (Zhang et al. 2019c). The differences in the composition of monosaccharides and their molar ratios in polysaccharides may be closely related to factors such as raw material species, isolation, and purification methods. Notably, mannose is the predominant component of CMP, whereas glucose is the main component of Se‐CMP. This shift suggests that Se influences the biosynthetic pathways of polysaccharide metabolites in *C. militaris* , leading to changes in structural composition.

### 3.3 FT‐IR

**FIGURE 3:**
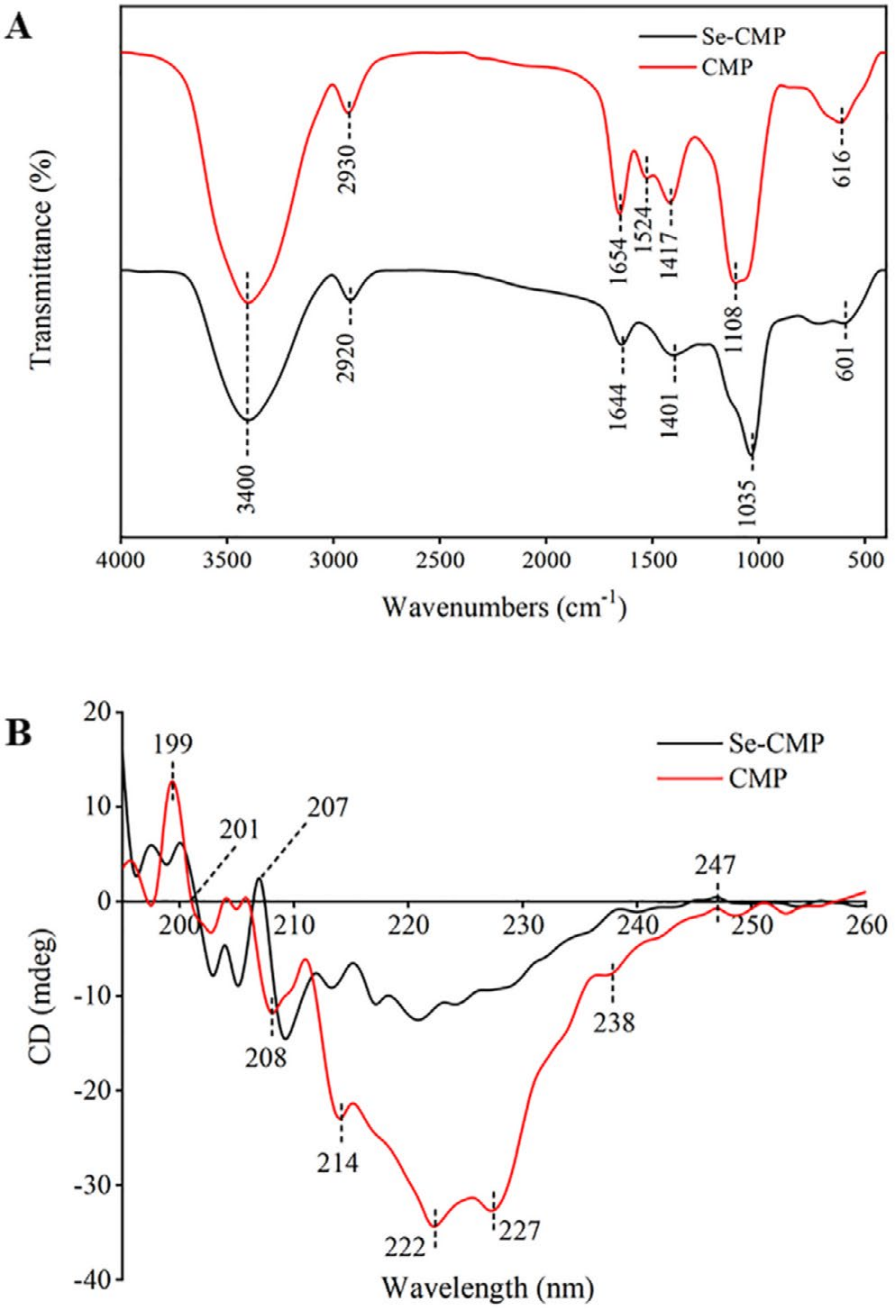
Fourier transform infrared spectrogram of CMP and Se‐CMP (A), Circular dichroism of CMP and Se‐CMP (B). CMP, *Cordyceps militaris* polysaccharides; Se‐CMP, selenium‐enriched *Cordyceps militaris* polysaccharides.

The FT‐IR spectra of CMP and Se‐CMP were observed from 4000 to 400 cm^−1^ (Figure 3A). Generally, the spectral profiles of both polysaccharides were strikingly similar, with close alignment of the AP of CMP and Se‐CMP. However, the absorption intensities of these two compounds differed slightly. This observation suggests that the structural frameworks of CMP and Se‐CMP remain similar despite Se enrichment. The broad and strong AP of CMP and Se‐CMP at 3400 cm^−1^ resulted from the stretching vibration of O‐H (Ji et al. 2018). The AP at 2920 and 2930 cm^−1^ was attributed to the C‐H stretching vibration of the −CH_2_ group (Zhang et al. 2018a); those near 1644 and 1654 cm^−1^ corresponded to the absorption of the carbonyl group C=O as part of the glycoside, and those at 1401 and 1417 cm^−1^ to the C‐H bending vibration (Liu et al. 2016). Furthermore, the presence of specific sharp peaks scattered throughout the 1000–1200 cm^−1^ range suggests the presence of the pyranose form of sugar residues (Xu et al. 2018). Se polysaccharides exist in various forms, including Se = O, O‐Se‐O, C‐O‐Se, and others (Gao et al. 2016). The distinctive AP at 601 and 1035 cm^−1^ corresponded to the stretching vibration of the selenium esters Se‐O‐C and O‐Se‐O bonds. Based on these results, Se and the polysaccharides were successfully connected through O‐Se‐O and Se‐O‐C bonds.

### 3.4 CD

The CD spectra revealed that both CMP and Se‐CMP exhibited negative and positive peaks within the wavelength range of 195–260 nm, suggesting the presence of compact triple‐helical structures (Figure 3B). A notable reduction in the areas of the positive and negative peaks was observed for Se‐CMP. This change indicates that the addition of Se renders the polysaccharides more flexible and reduces the symmetry of sugar chains within the polysaccharide molecules (Yan et al. 2012).

### 3.5 Se‐CMP Alleviates IR In Vitro

#### 3.5.1 α‐Glucosidase Inhibitory Activity

Figure S1 illustrates the inhibitory effects of Se‐CMP on α‐glucosidase activity. Se‐CMP affected α‐glucosidase activity in a dose‐dependent manner. The half‐maximal inhibitory concentration (IC_50_) of Se‐CMP on α‐glucosidase was 0.474 mg/mL, indicating the potent inhibition of α‐glucosidase.

#### 3.5.2 Effects on Cell Viability and Morphology

**FIGURE 4:**
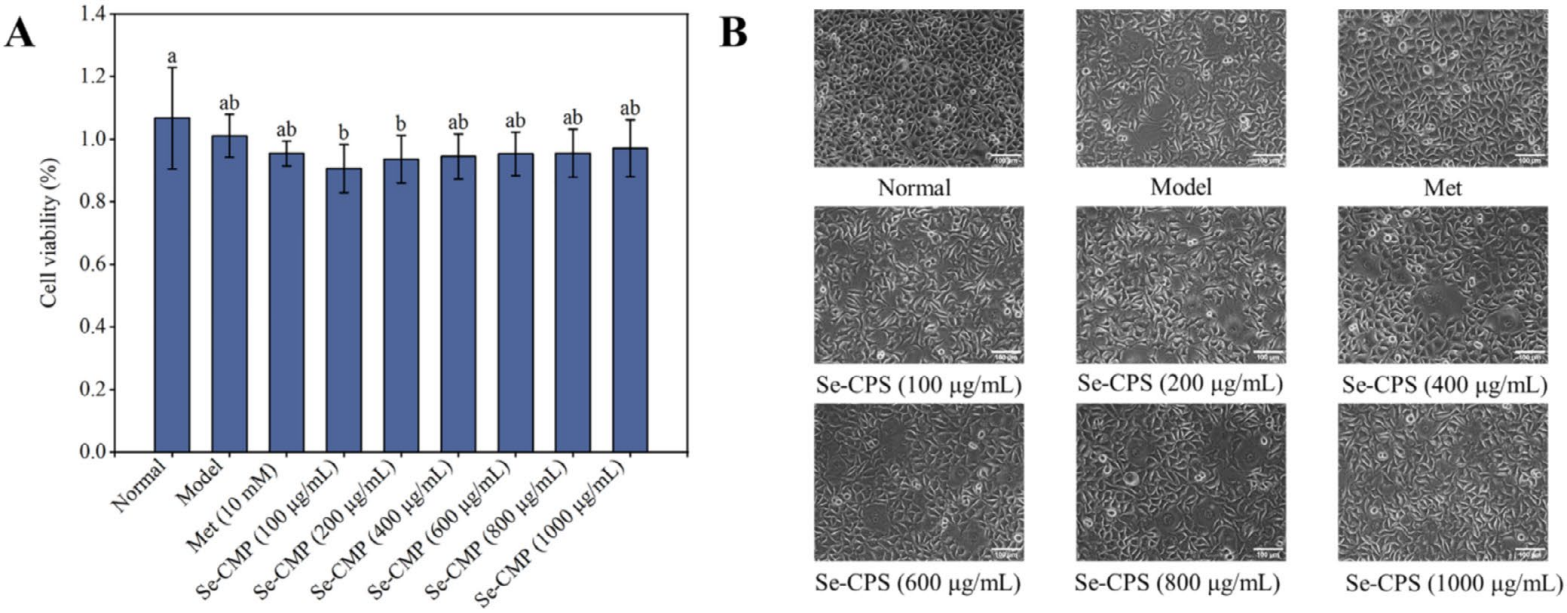
Examination of the effect of Se‐CMP in IR HepG2 cell viability. (A) Cell morphology of HepG2 cells at 200× magnification. (B) Normal, normal control group; Met, metformin group; Model, insulin‐resistant group; Se‐CMP, selenium‐enriched *Cordyceps militaris* polysaccharides. Data marked with different letters are significantly different at *p* < 0.05.

The cytotoxic effect of Se‐CMPs on IR HepG2 cells was investigated using the MTT assay. Se‐CMP treatment of IR HepG2 cells did not significantly alter cell viability compared to that in the model group (*p* > 0.05), confirming that Se‐CMP was safe for IR HepG2 cells (Figure 4A). Compared to the normal control group (Figure 4B), the cells in the model group were ruptured and shrunken, with a reduced number of cells and irregular shapes, showing the typical morphology of islet damage. As the sample concentration increased, the number of cells gradually increased, the degree of cell rupture was alleviated, and the shape of the cells gradually became regular, returning to the normal cell state. At the highest concentration of 1000 μg/mL, there was no obvious distinction between the experimental group and the normal control and Met groups. The findings indicate that Se‐CMP has a reparative effect on IR HepG2 cells.

#### 3.5.3 Glucose Consumption and Intracellular Glycogen Content in IR HepG2 Cells

**FIGURE 5:**
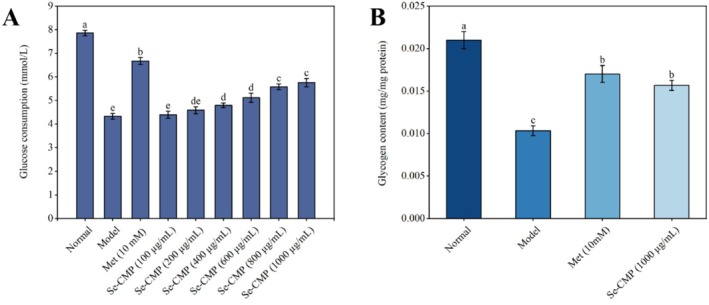
Examination of the effect of Se‐CMP on glucose consumption in IR HepG2 cells (A) and the glycogen content in IR HepG2 cells (B). Normal, normal control group; Met, metformin group; Model, insulin‐resistant group; Se‐CMP, selenium‐enriched *Cordyceps militaris* polysaccharides. Data marked with different letters are significantly different at *p* < 0.05.

Glucose consumption in the insulin‐treated model group was significantly lower than that in the control group (*p* < 0.05), confirming the validity of the experimental model (Figure 5A). Se‐CMP‐treated HepG2 cells exhibited a concentration‐dependent increase in glucose consumption. Treatment with 1000 μg/mL Se enhanced the glucose consumption of IR HepG2 cells to 5.87 ± 0.20 mg/mg, indicating that Se‐CMP attenuated IR in HepG2 cells (*p* < 0.05). Based on the results of Se‐CMP on glucose consumption and cell morphology of IR HepG2 cells, 1000 μg/mL Se‐CMP was chosen for subsequent analyses in this study. Se‐CMP significantly increased glycogen content in IR HepG2 cells compared to the model group (*p* < 0.05). The glycogen content was similar to that in the Met group (*p* > 0.05) (Figure 5B). These results demonstrate that Se‐CMP alleviates IR by inducing IR‐HepG2 cells to absorb and utilize glucose.

#### 3.5.4 Levels of ROS, SOD, CAT, and GSH‐Px in IR HepG2 Cells

**FIGURE 6:**
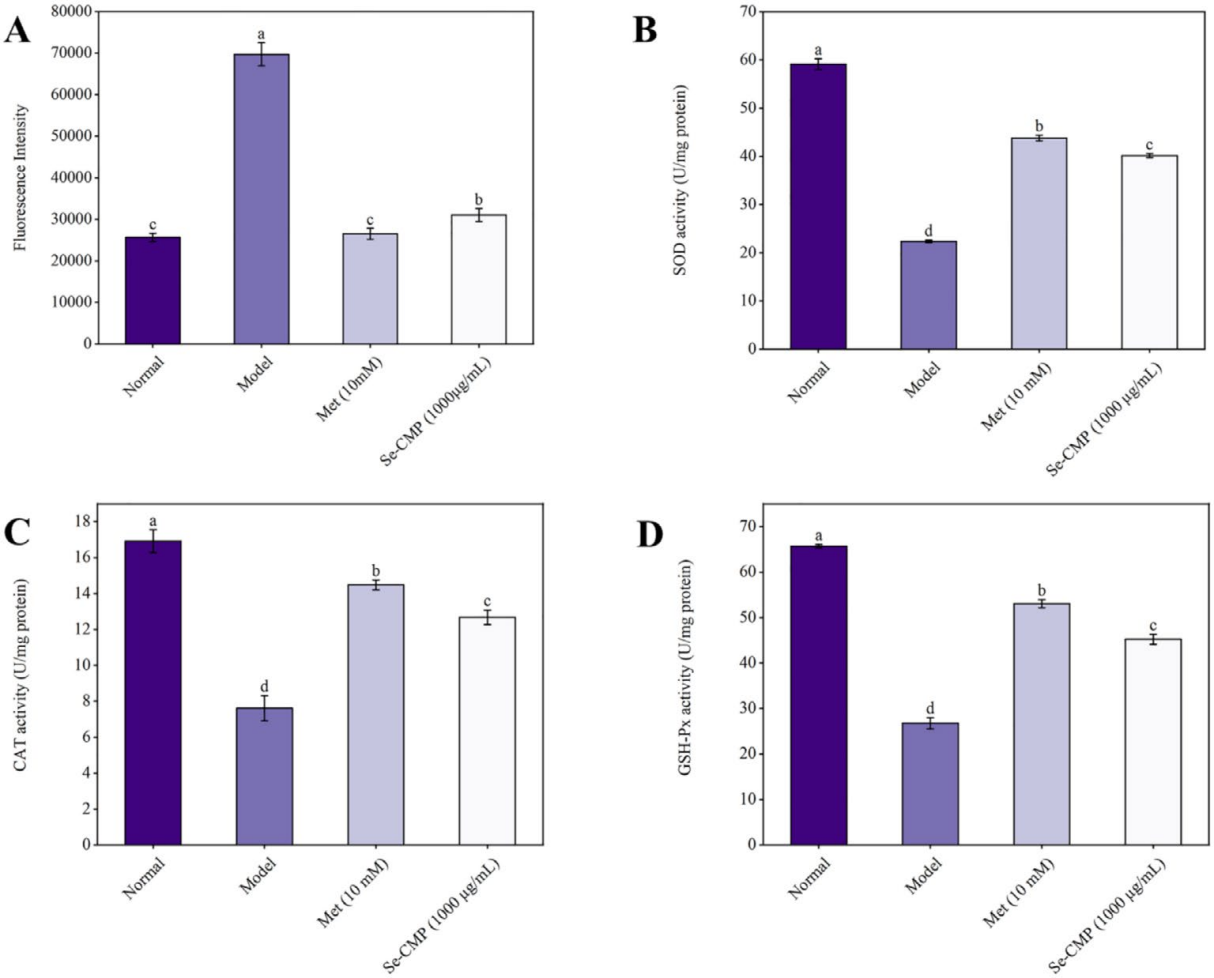
Effects of Se‐CMP on the ROS generation (A), and the activities of SOD (B), CAT (C), and GSH‐Px (D) in IR HepG2 cells. CAT, catalase; GSH‐Px, glutathione peroxidase; Met, metformin group; Model, insulin‐resistant group; Normal, normal control group; ROS, reactive oxygen species; Se‐CMP, selenium‐enriched *Cordyceps militaris* polysaccharides; SOD, superoxide dismutase. Data marked with different letters are significantly different at *p* < 0.05.

Compared to the control group, the model group showed a significant increase in ROS generation (*p* < 0.05; Figure 6A). Conversely, ROS generation was significantly lower in the Se‐CMP‐treated group than in the model group (*p* < 0.05). Furthermore, the activities of SOD, CAT, and GSH‐Px were significantly reduced in the model group compared to those in the normal control group (*p* < 0.05; Figure 6B,C,D). Se‐CMP treatment alleviated this reduction (*p* < 0.05), indicating its effectiveness in mitigating oxidative stress (OS) in IR HepG2 cells.

#### 3.5.5 Se‐CMP Activates the PI3K/AKT/ GLUT4 Signal Pathway in IR HepG2 Cells

**FIGURE 7:**
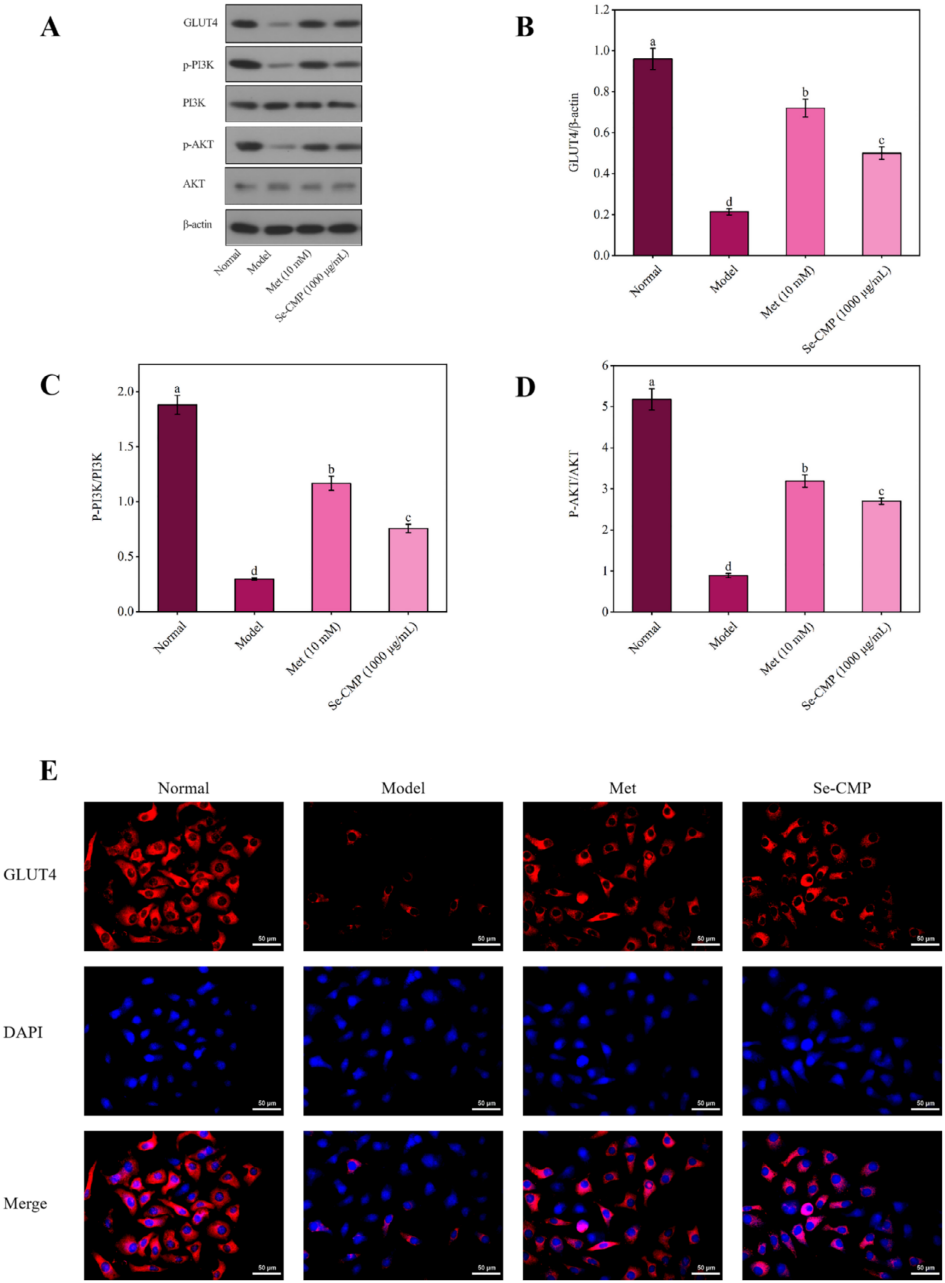
The expression variations of GLUT4, p‐PI3k/PI3K, p‐AKT/AKT in cells were determined by Western blot, with β‐actin serving as a loading control (A), and comparison of the levels of the phosphorylated protein relative to their non‐phosphorylated counterparts in gray scale (B–D). Immunofluorescence staining image of Se‐CMP‐treated IR HepG2 cells (E). AKT, protein kinase B; GLUT4, glucose transporters 4; Met, metformin; Model, insulin‐resistant group; Normal, normal control group; PI3K, phosphatidylinositol 3‐kinase; Se‐CMP, selenium‐enriched *Cordyceps militaris* polysaccharides. Data marked with different letters are significantly different at *p* < 0.05.

The expressions of GLUT4, p‐PI3K, and p‐AKT were downregulated in the model group compared to the normal control group (all *p* < 0.05), suggesting the presence of IR in HepG2 cells (Figure 7A–D). Se‐CMP treatment significantly increased the expressions of p‐AKT, GLUT4, and p‐PI3K compared to that in the model group (all *p* < 0.05). The expression of GLUT4 was examined by immunofluorescence staining to validate its involvement in the absorption of glucose induced by Se‐CMP. Se‐CMP treatment increased the expression of GLUT4 in cells and promoted the translocation of GLUT4 from the vesicles to the cell membrane compared with the model group (Figure 7E). These results suggest that Se‐CMP treatment alleviated IR in HepG2 cells, which might be correlated with the PI3K/AKT signaling pathway.

## 4 Discussion

**FIGURE 8:**
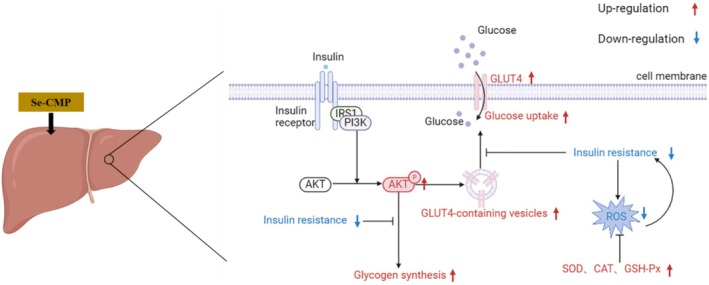
Diagram of the mechanism by which selenium‐enriched *Cordyceps militaris* polysaccharides alleviate insulin resistance in HepG2 cells. AKT, protein kinase B; CAT, catalase; GLUT4: glucose transporters 4; GSH‐Px, glutathione peroxidase; PI3K, phosphatidylinositol 3‐kinase; ROS, reactive oxygen species; Se‐CMP, selenium‐enriched *Cordyceps militaris* polysaccharides; SOD, superoxide dismutase.

In this study, the physicochemical properties of CMP and Se‐CMP were compared, and the mechanisms underlying Se‐CMP‐mediated alleviation of IR were investigated. Se content, molecular weight, monosaccharide composition, UV, FT‐IR, and CD analyses confirmed Se‐CMP as a non‐homogeneous and neutral polysaccharide. Se‐CMP effectively mitigated insulin‐induced adverse effects on glucose metabolism by enhancing the expression and translocation of GLUT4, potentially through the activation of the PI3K/AKT/GLUT4 signaling pathway. Additionally, Se‐CMP alleviated OS and promoted normal insulin signaling by reducing ROS levels and enhancing the activities of the antioxidant enzymes SOD, CAT, and GSH‐Px (Figure 8). These properties highlight the therapeutic potential of Se‐CMPs in the management of glucose metabolism disorders.

Metal ions can be used as nutritional supplementation interventions to play an adjunctive role in the pharmacological treatment of T2DM (Ye et al. 2019). Se, as an insulin mimetic, can activate key proteins in the insulin signaling cascade response (Stapleton 2000). Se also has antioxidant properties, scavenging free radicals and mitigating oxidative damage. Therefore, Se supplementation in appropriate amounts can alleviate IR and lower blood glucose (Ogawa‐Wong et al. 2016). In this study, Se nanoparticles were used as raw material enriched within *Cordyceps militaris* to investigate the anti‐IR effects of Se‐CMP. However, it remains to be elucidated whether the difference in Se source would cause a difference in Se‐CMP bioactivity.

α‐Glucosidase, which is primarily located on the brush border of small intestinal mucosal cells, hydrolyzes glycosidic bonds and degrades oligosaccharides to monosaccharides (Sun et al. 2021). Inhibition of the activity of this enzyme affects glucose conversion and lowers blood glucose (Cao et al. 2019; Kim et al. 2010). The assay for α‐glucosidase inhibitory activity is widely utilized to assess the hypoglycemic action of bioactive substances in vitro. For instance, *C. militaris* exopolysaccharides III inhibit α‐glucosidase, with a positive correlation of the inhibition rate with sample concentration, peaking at 55.94% ± 1.34% at 3 mg/mL (Sun et al. 2021). In the present study, the inhibition of α‐glucosidase by CMP increased with the increase in concentration, with an IC_50_ of 0.474 mg/mL, which is markedly lower than the IC_50_ reported elsewhere (Zhu et al. 2014). This finding indicates that CMP has an inhibitory effect on α‐glucosidase and that the enrichment of Se in polysaccharides can enhance the inhibitory effect on α‐glucosidase.

As the largest metabolic regulator in the human body, the liver plays a pivotal role in glucose homeostasis through the uptake and utilization of glucose to decrease blood glucose levels and convert glucose into hepatic glycogen for storage. HepG2 is a human hepatoma cell line with hepatic properties such as lipid metabolism, glycogen synthesis, and insulin signaling (Wang et al. 2025). Therefore, HepG2 cells are considered a suitable cell model for studying IR and antioxidant functions (Cai et al. 2015; Wang et al. 2025). In this study, an IR HepG2 cells model was established by culturing HepG2 cells with insulin. This model is instrumental in understanding hepatocyte metabolic dysregulation, which is crucial for the onset of IR.

Glycogen is a multibranched glucose polysaccharide and is the main form of energy storage in humans. Gluconeogenesis and glycogenolysis are major pathways involved in hepatic glucose metabolism (Deng et al. 2020). Regulating the levels of glycogen and key enzymes in glucose metabolism helps improve the function of IR HepG2 cell glucose metabolism, which can effectively alleviate the symptoms of hyperglycemia and reduce the incidence of diabetes mellitus and its complications (Tang et al. 2015). In this study, Se‐CMP treatment significantly increased IR HepG2 cells glucose consumption and intracellular glycogen content, thereby reducing IR. Similar to this work, Anemarrhena asphodeloides heteropolysaccharides and Sargassum pallidum polysaccharides markedly increase the activities of pyruvate kinase and hexokinase, as well as glucose consumption and glycogen content in IR HepG2 cells, demonstrating their potential in therapeutic applications (Cao et al. 2019; Sun et al. 2021).

The GLUT family comprises a series of transmembrane proteins that have crucial roles in regulating glucose transport across cell membranes. Of the 14 identified GLUT members, GLUT4 is the sole insulin‐sensitive transporter. In the presence of insulin, GLUT4 is dynamically mobilized from intracellular vesicles to the cell surface, enhancing cellular glucose uptake. The PI3K/AKT signaling pathway is also essential for mediating the effects of insulin. This pathway regulates various aspects of glucose metabolism, including glucose transport, glycogen synthesis, and inhibition of gluconeogenesis (He et al. 2007). PI3K binds to phosphorylated insulin receptor substrate 1 (IRS1) on the cell membrane surface and activates downstream AKT, which translocates GLUT4 to the plasma membrane from intracellular storage vesicles for glucose transport via a downstream signaling cascade. This process has a central role in the maintenance of glucose homeostasis (Xu et al. 2018). Under IR conditions, activation of the PI3K/AKT pathway is impaired. This injury is characterized by decreased AKT phosphorylation and activation. The resultant inefficiency in GLUT4 translocation leads to decreased glucose uptake by cells and, consequently, increased blood glucose levels. Thus, understanding the regulatory mechanisms and disruptions within this signaling pathway is vital for the development of targeted therapies for diabetes and other metabolic disorders characterized by IR. This study revealed that Se‐CMP treatment significantly enhanced the expression of p‐AKT, GLUT4, and p‐PI3K while promoting GLUT4 translocation from intracellular vesicles to the plasma membrane in IR HepG2 cells. These may promote glucose utilization and alleviate IR. Glucans from *Euryale ferox Salisb*. *Seeds* promote glucose uptake and alleviate IR by increasing the expression of GLUT4, p‐AKT, AKT, PI3K, and IRS (Zhang et al. 2019b). *C. cicadae* polysaccharides improve glucose utilization and IR by activating the PI3K/AKT signaling pathway, which involves GLUT4, AKT, IRS‐1, and PI3K (Wang et al. 2023). *Nitraria tangutorum Bobr*. ameliorates insulin sensitivity and glucose metabolism disorders by regulating the IRS1/PI3K/AKT signaling pathway and the expressions of its downstream targets GLUT4, FOXO1, and GSK3β (Jiang et al. 2023).

OS is increasingly being recognized as being pivotal in the pathogenesis of diabetes and its complications (Ježek et al. 2019). This results from an imbalance between ROS generation and the ability of the antioxidant system to remove substances (Li et al. 2024), which leads to a cascade of pathological reactions. Specifically, in the IR state, OS impaired the oxidative phosphorylation capabilities of liver cell mitochondria. This impairment is connected to an increase in fatty acid β‐oxidation within hepatocytes, contributing to lipid overload and an escalation in peroxidation products (Rehman and Akash 2017; Wang et al. 2025). Such changes negatively affect the phosphorylation of the insulin receptor and insulin receptor substrate, diminish their influence on downstream PI3K, and disrupt normal insulin signaling pathways. Consequently, these disruptions can exacerbate the symptoms of DM and perpetuate a deleterious cycle (Huang et al. 2018). Normally, mitochondrial SOD degrades anionic superoxide to hydrogen peroxide, which is then converted into water and oxygen by CAT (Yaribeygi et al. 2019). GSH‐Px is another crucial enzyme that facilitates the catabolism of peroxides and is ubiquitously present in organisms. SOD, CAT, and GSH‐Px work together to protect cells from OS by converting ROS into harmless substances. Therefore, scavenging ROS in the body and increasing the activities of CAT, GSH‐Px, and SOD enzymes are important for reducing the damage caused by OS, thus slowing the onset and progression of DM and its associated complications. In this study, Se‐CMP treatment markedly decreased the levels of ROS and significantly enhanced the activities of CAT, GSH‐Px, and SOD in IR HepG2 cells. Similarly, another study reported that selenylated *Momordica charantia L*. polysaccharides significantly increased the activities of SOD, CAT, and GSH‐Px and significantly reduced MDA levels in the liver, thereby attenuating OS in streptozotocin‐induced diabetic mice (Ru et al. 2020). However, the present study only confirmed the role of Se‐CMP in improving IR at the cellular level. Future studies need to further validate its efficacy at the animal model level and combine it with multi‐omics approaches to deeply resolve the molecular mechanisms and signaling pathway network of Se‐CMP in alleviating IR.

## 5 Conclusions

In this study, Se‐CMP was produced through biotransformation, and its physicochemical and alleviated IR functions were examined. The alleviated IR action of Se‐CMP is proposed to occur through activation of the PI3K/AKT/GLUT4 signaling pathway and reduction of levels of OS. However, the alleviated IR effect of Se‐CMPs and its underlying mechanisms must be further verified in vivo. These preliminary findings suggest the potential of Se‐CMP as a functional food supplement for the management of T2DM, offering a natural adjunct to traditional treatment modalities.

## Author Contributions

**Jiali Chen:** data curation (equal), methodology (equal), software (equal), visualization (equal), writing – original draft (equal). **Xiumin Zhang:** conceptualization (equal), formal analysis (equal), investigation (equal). **Yanting Chang:** methodology (equal), software (equal), validation (equal). **Na Zhang:** conceptualization (equal), data curation (equal), project administration (equal). **Xiqing Yue:** funding acquisition (equal), supervision (equal), writing – review and editing (equal). **Mohan Li:** conceptualization (equal), data curation (equal), investigation (equal), project administration (equal), resources (equal), supervision (equal), writing – review and editing (equal). **Wentao Jia:** project administration (equal), resources (equal), supervision (equal), validation (equal), writing – review and editing (equal).

## Conflicts of Interest

The authors declare no conflicts of interest.

## Supporting information


**Figure S1.** Inhibition on α‐glucosidase ability of Se‐CMP. Se‐CMP, selenium‐enriched *Cordyceps militaris* polysaccharides.

**Table S1.** Results of high‐performance gel permeation chromatographic analysis of CMP.
**Table S2.** Results of high‐performance gel permeation chromatographic analysis of Se‐CMP.
**Table S3.** Monosaccharide composition of CMP and Se‐CMP.

## Data Availability

Data will be made available on request.
