# Supplementary material for: Selenium‐Enriched Cordyceps militaris Polysaccharides Alleviate Insulin Resistance in HepG2 Cells by Regulating the PI3K/AKT/GLUT4 Signaling Pathway
Source: Food Sci Nutr. 2025 May 10;13(5):e70246. doi: 10.1002/fsn3.70246 (PMC12064949; doi:10.1002/fsn3.70246)
Supplement: Supplementary file 1 — Figure S1. Inhibition on α‐glucosidase ability of Se‐CMP. Se‐CMP, selenium‐enriched Cordyceps militaris polysaccharides. [file FSN3-13-e70246-s002.docx]

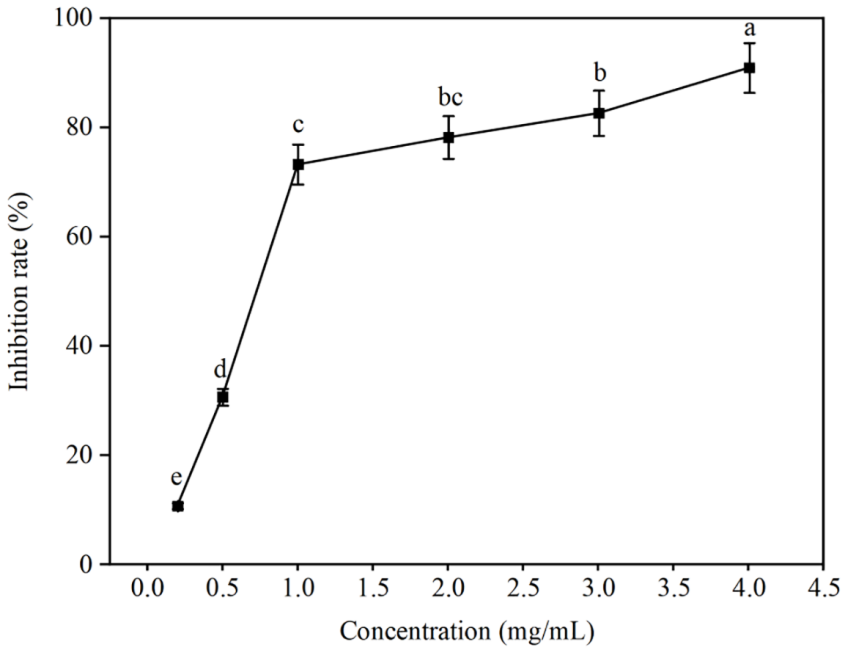


**Supplementary Fig. 1.** Inhibition on α-glucosidase ability of Se-CMP. Se-CMP: selenium-enriched *Cordyceps militaris* polysaccharides.
