# Supplementary material for: Selenium‐Enriched Cordyceps militaris Polysaccharides Alleviate Insulin Resistance in HepG2 Cells by Regulating the PI3K/AKT/GLUT4 Signaling Pathway
Source: Food Sci Nutr. 2025 May 10;13(5):e70246. doi: 10.1002/fsn3.70246 (PMC12064949; doi:10.1002/fsn3.70246)
Supplement: Supplementary file 2 — Table S1. Results of high‐performance gel permeation chromatographic analysis of CMP. Table S2. Results of high‐performance gel permeation chromatographic analysis of Se‐CMP. Table S3. Monosaccharide composition of CMP and Se‐CMP. [file FSN3-13-e70246-s001.docx]

**Supplementary Table 1.** Results of high-performance gel permeation chromatographic analysis of CMP.

| RT (min) | Mw (Da) | Mp (Da) | Mn (Da) | PAR(%) | Mw/Mn |
| --- | --- | --- | --- | --- | --- |
| 39.835 | 26881 | 22035 | 18196 | 67.12 | 1.48 |
| 41.783 | 12749 | 10833 | 8978 | 24.85 | 1.42 |
| 45.405 | 3185 | 2893 | 2414 | 8.03 | 1.32 |

RT: retention time, Mw: weight average molecular weight, Mp: peak molecular weight, Mn: number average molecular weight, PAR: peak area ratio, Mw/Mn: distribution coefficient. The regression equation is lg Mw =-0.1663x + 11.054 (R^2^=0.9950), lg Mp =-0.1583x + 10.649 (R^2^=0.9911),lg Mn =-0.1575x + 10.534 (R^2^=0.9937 ). CMP, *Cordyceps militaris* polysaccharides; Se-CMP, selenium-enriched *Cordyceps militaris* polysaccharides.

**Supplementary Table 2.** Results of high-performance gel permeation chromatographic analysis of Se-CMP.

| RT (min) | Mw (Da) | Mp (Da) | Mn (Da) | PAR(%) | Mw/Mn |
| --- | --- | --- | --- | --- | --- |
| 27.540 | 2979189 | 1947233 | 1571991 | 23.66 | 1.90 |
| 30.655 | 903802 | 625624 | 507969 | 21.24 | 1.78 |
| 33.650 | 287081 | 209993 | 171445 | 3.16 | 1.67 |
| 40.362 | 21968 | 18184 | 15031 | 46.41 | 1.46 |
| 45.670 | 2878 | 2627 | 2193 | 5.53 | 1.31 |

RT: retention time, Mw: weight average molecular weight, Mp: peak molecular weight, Mn: number average molecular weight, PAR: peak area ratio, Mw/Mn: distribution coefficient. The regression equation is lg Mw =-0.1663x + 11.054 (R^2^=0.9950), lg Mp =-0.1583x + 10.649 (R^2^=0.9911),lg Mn =-0.1575x + 10.534 (R^2^=0.9937 ). CMP, *Cordyceps militaris* polysaccharides; Se-CMP, selenium-enriched *Cordyceps militaris* polysaccharides.

**Supplementary Table 3.** Monosaccharide composition of CMP and Se-CMP.

| Sample | Molar ratios | | | | | |
| --- | --- | --- | --- | --- | --- | --- |
|  | Arabinose | Glucosamine hydrochloride | Galactose | Glucose | Xylose | Mannose |
| CMP | 0.041 | 0.001 | 0.313 | 0.226 | 0.059 | 0.360 |
| Se-CMP | 0.022 | 0.001 | 0.133 | 0.659 | 0.036 | 0.150 |

CMP, *Cordyceps militaris* polysaccharides; Se-CMP, selenium-enriched *Cordyceps militaris* polysaccharides.
